# Snapshots of archaeal DNA replication and repair in living cells using super-resolution imaging

**DOI:** 10.1093/nar/gky829

**Published:** 2018-09-13

**Authors:** Floriane Delpech, Yoann Collien, Pierre Mahou, Emmanuel Beaurepaire, Hannu Myllykallio, Roxane Lestini

**Affiliations:** Laboratoire d’Optique et Biosciences, Ecole Polytechnique, CNRS UMR7645 – INSERM U1182, 91128 Palaiseau Cedex, France

## Abstract

Using the haloarchaeon *Haloferax volcanii* as a model, we developed nascent DNA labeling and the functional GFP-labeled single-stranded binding protein RPA2 as novel tools to gain new insight into DNA replication and repair in live haloarchaeal cells. Our quantitative fluorescence microscopy data revealed that RPA2 forms distinct replication structures that dynamically responded to replication stress and DNA damaging agents. The number of the RPA2 foci *per* cell followed a probabilistic Poisson distribution, implying hitherto unnoticed stochastic cell-to-cell variation in haloarchaeal DNA replication and repair processes. The size range of haloarchaeal replication structures is very similar to those observed earlier in eukaryotic cells. The improved lateral resolution of 3D-SIM fluorescence microscopy allowed proposing that inhibition of DNA synthesis results in localized replication foci clustering and facilitated observation of RPA2 complexes brought about by chemical agents creating DNA double-strand breaks. Altogether our *in vivo* observations are compatible with earlier *in vitro* studies on archaeal single-stranded DNA binding proteins. Our work thus underlines the great potential of live cell imaging for unraveling the dynamic nature of transient molecular interactions that underpin fundamental molecular processes in the Third domain of life.

## INTRODUCTION

In the three domains of life, DNA is replicated by dynamic multiprotein machines called replisomes that couple the activities of several proteins required for copying genetic information. To fully understand how this crucial and highly efficient process occurs, information on the intracellular organization of the replisomes is required. The spatiotemporal localization and dynamics of intracellular replisomes have been extensively investigated in Bacteria and Eukarya using functional fluorescent derivatives of replisome components. These studies have revealed that DNA replication and synthesis of nascent DNA occur at discrete sites within the cell that can be localized through formation of stable fluorescent replication foci (RF). Obtaining analogous information on the organization of archaeal DNA replication in living cells would be of great interest from a mechanistic and evolutionary point of view, as archaeal chromosomes are circular and replicated either using single (1) or multiple (2) replication origins by a protein machinery resembling that of eukaryotes (3). The structure and length of short archaeal replication intermediates are also very similar to those of eukaryotic Okazaki fragments (4), further attesting to the close relationship between archaeal and eukaryotic DNA replication processes.

In Bacteria, fluorescent versions of several replisome components, including the replication clamp (DnaN) and the single-stranded DNA binding (SSB) proteins, have been used to localize and quantify replisomes in live cells. In *Bacillus subtilis* and *Caulobacter crescentus*, the replisome localization patterns indicated that the two sister replication forks form a stable focus and spatially co-localize throughout the replication cycle (5,6). By contrast, in *Escherichia coli*, each sister fork may act independently and replisomes move along the DNA template from the origin to the terminus (7–9). More recent imaging experiments combined with statistically robust image analyses have suggested that both in *E. coli* and *B. subtilis* two replication forks originating from a single replication origin co-localize up to 80% of the replication cycle, although occasional separation of sister forks is also possible (10).

Advanced optical microscopy techniques have been used to investigate the replisome localization and dynamics also in eukaryotes (11,12). These studies have underlined how technical developments in optical microscopy methods beyond the Abbe (diffraction) limit have changed our views on the intranuclear organization of DNA replication. In particular, improved lateral or axial resolution of stimulated emission depletion (STED) and 3D-structured illumination (3D-SIM) microscopy techniques was necessary to detect and quantify up to 6000 RF in the nucleus of human cells (13,14). Both super-resolution techniques revealed independently that the diameter of the eukaryotic RF varies between 40 and 210 nm with an average value of ≈150 nm. This size estimation was in close agreement with previous electron microscopy studies (15). The number of detected RF is fully consistent with the length of the S-phase and genome size of human and mouse cells (16). Several studies have suggested that these RF may reflect the association of neighboring replicons (17) and may correspond to replication domains that carry, on average, four co-replicating DNA regions of approximately 20 kb in length (18).

Studies on the intracellular localization of DNA synthesis in archaeal cells are scarce, as this topic has only been addressed in *Sulfolobus* species (19). In these species and possibly other Crenarcheota, the vast majority of the cells contained two to three peripherally located replication foci detected either by PCNA1 antibodies or click-labeling of alkyne (EdU) labeled nascent DNA. *Sulfolobus* chromosomes contain three replication origins *per* circular chromosome of ∼3 Mb (2), suggesting that the observed foci may correspond to DNA replication structures directly or indirectly interacting with the cell membrane. This study also suggested that the sister replication forks established at specific origins remained in close vicinity (within the diffraction limit), while forks initiating from distinct further located origins remained spatially separated. More recently it was demonstrated that in *Sulfolobus* species, viral DNA synthesis also occurs near the periphery of the cell infected by a SIRV2 virus (20).

To gain new insight into DNA replication in living archaeal cells, we turned our attention to the salt-loving euryarchaeon *Haloferax volcanii*, as the mesophilic and aerobic lifestyle of this halophile facilitates the use of GFP fusions to study intracellular protein localization and dynamics (21). This species carries one main chromosome (3483 kb) with several replication origins and a high ploidy number (up to 30), as well as two smaller megaplasmids (22–24). The potential number of simultaneously active replication origins and/or replicons is thus very high for this prokaryotic species. Moreover, its chromatin architecture is similar to that of eukaryotes (25), as archaeal histone homodimers are capable of wrapping DNA with a periodicity of 70–80 nucleotides *in vivo* and *in vitro* (25,26). These characteristics clearly make *H. volcanii* a very interesting model for understanding the DNA replication of several replicons using fluorescence microscopy.

We successfully constructed an *H. volcanii* strain expressing from the native chromosomal locus the functional single-stranded DNA binding protein (RPA2) fused to Green Fluorescent Protein (GFP). Together with nascent DNA labeling, the obtained GFP::RPA2 fusion protein was an ideal proxy for haloarchaeal DNA replication and repair studies. Using wide-field and SIM super-resolution fluorescence microscopy coupled with quantitative image analysis, we demonstrated that RPA2 forms distinct RF in proliferating cells that actively responded to replication stress and DNA damaging agents. The number of detected RF *per* individual cell was surprisingly low and demonstrated a significant cell-to-cell variation influenced by stochastic processes.

## MATERIALS AND METHODS

### Chemicals

Unless stated otherwise, all chemicals used were purchased from Sigma-Aldrich.

### Molecular biology techniques

Isolation of genomic and plasmid DNA and transformation of *H. volcanii* were carried out using published protocols (27–29). Standard molecular biology techniques were used for DNA isolation and manipulation. All PCR reactions were performed using Q5 polymerase from New England Biolabs (Ipswich, Massachusetts, USA) All other enzymes were purchased from Thermo Fisher Scientific (Villebon-sur-Yvette, France).

### Strains, plasmids and growth conditions

*Escherichia coli* strains XL1-Blue MRF’ (Δ*mcrA183* Δ*mcrCB-hsdSMR-mrr173 endA1 supE44 thi-1 recA1* gyrA96 relA1 lac*[F′* proAB lacI^q^*ZΔ*M15*Tn*10*]*) and GM121 (F^−^*dam^−^3 dcm^−^6 ara^−^14 fhuA31 galK2 galT22 hdsR3 lacY1 leu-6 thi-1 thr-1 tsx-78*) were used for cloning. The latter *dam*^−^*dcm*^−^ strain was also used to prepare unmethylated plasmid DNA for efficient transformation of *H. volcanii. E. coli* transformants were selected on LB plates containing 100 μg*/*ml ampicillin, 0.5 mM IPTG and 80 μg/ml X-Gal.

*H. volcanii* H26 strain (Δ*pyrE2 rpa2^+^*) was used during this work (28). *H. volcanii* cultures using enriched Hv-YPC or Hv-Ca media were grown at 45°C, as described previously (27). Hv-YPC contained (per liter) 144 g of NaCl, 21 g of MgSO_4_.7H_2_O, 18 g of MgCl_2_.6H_2_O, 4.2 g of KCl, and 12 mM Tris–HCl (pH 7.5), Yeast extract (0.5%, wt/vol; Difco), 0.1% (wt/vol) peptone (Oxoid), and 0.1% (wt/vol) Casamino Acids (Difco). Casamino Acids medium (Hv-Ca) was similar to Hv-YPC except that yeast extract and peptone were omitted, and Casamino Acids were added to a final concentration of 0.5% (wt/vol). Different drugs were added after 2 hours of growth at 45°C to over-night cultures that were diluted to OD_600_ ≈ 0.1, and incubation was continued for 18 h. Where indicated, different compounds were added to liquid cell cultures at the following concentrations: aphidicolin [2.5, 5, 10 or 20 μg/ml dissolved in 100% dimethyl sulfoxide (DMSO)] and phleomycin (12.5, 25, 50, 75 or 100 μg/ml). When aphidicolin stock solution was added to cells, only DMSO was added to control cells. To determine the fraction of surviving cells, cultures were diluted in 18% Salt Water (18% SW) containing 2.5 M NaCl, 90 mM MgCl_2_.6H_2_O, 90 mM MgSO_4_.7H_2_O, 60 mM KCl, 10 mM Tris–HCl pH 7.5. Aliquots of 20 μl were spotted on Hv-YPC plates and individual colonies were counted after 4 days incubation at 45°C.

### Construction of *gfp*::*rpa2* strain

First, the fusion plasmid pFD2 carrying *gfp*^+^::*rpa2*^+^ allele was constructed. Sequences of all oligonucleotides used during this work are indicated in the supplementary Supplementary Table S1. Three PCR products were amplified using genomic DNA from strain H26 (Δ*pyrE2*): (i) the up-stream region of *rpa2* using primers RL129 and RL130, (ii) the *gfp* gene using primers RL85bis and RL124 on pRL29 plasmid DNA (21) and (iii) the first 400 bp of *rpa2* gene using primers RL131 and RL132. RL130 and RL131 both contain in their 5′ extremity 18-based homology to the *gfp* PCR fragment (underlined in the Supplementary Table S1). Final PCR reaction was performed by mixing three PCR fragments (100 ng of each) and using RL129 and RL132 as primers. A PCR fragment containing US-*gfp*^+^-*rpa2*^+’^ was recovered and cloned into the pTA131 plasmid *(pyrE2*^+^). For this purpose, the pTA131 plasmid (27) was linearized by EcoRV digestion and the blunt extremities dephosphorylated by Antarctic Phosphatase. The US-*gfp*^+^-*rpa2*^+’^ fragment was phosphorylated using T4 polynucleotide kinase prior to ligation with pTA131 and transformation in *E. coli* XL-1 blue. The presence of the correct insert in the plasmid of white colonies was tested using colony PCR with primers BSF2 and BSR3. The sequence of one selected plasmid, pFD2, was further confirmed by sequencing on both strands. Then pFD2 was used to transform H26 strain (Δ*pyrE2*) using the pop-in/pop-out method as described previously (28). The presence of the *gfp* gene was tested by PCR on ten colonies from the pop-out plates using primers RL85bis and RL124. Two out of ten contained the *gfp* gene. Finally, the insertion of the *gfp*^+^::*rpa2*^+^ allele at the locus was further confirmed by amplification of the locus using primers RL141) and RL165. A PCR product of 2996 bp length was obtained for the two *gfp*^+^::*rpa2*^+^ clones, while a product of 2246 bp was obtained in the control performed on H26 DNA. Note that using an analogous approach we were unable to obtain constructs expressing chromosomally encoded GFP::PCNA or PCNA::GFP fusion proteins

### Construction of Δ*hts* Δ*hdrB* strain

To generate the Δ*hts* Δ*hdrB* construct (because both genes are in an operon), the upstream region (US) of *hts* and the down-stream region (DS) of *hdrB* were cloned into pTA131 to generate the pRL55 plasmid used to construct the deletion strain by a gene knockout system (28). The US region was generated by PCR on H26 genomic DNA (Δ*pyrE2*) using primers RL208 and RL209. The DS region was generated by PCR on H26 genomic DNA using primers RL210 and RL211. Each PCR product contained 30 bp homology with adjacent fragments for sequence and ligation independent cloning (SLIC): (i) with the pTA131 linearized fragments after NotI and EcoRI double-digestion (contained in RL208), and with the DS fragment (contained in RL209) in the US fragment, and (ii) with the US fragment (contained in RL210), and with the pTA131 (27) linearized fragments after NotI and EcoRI double-digestion (contained in RL211) in the DS fragment. Following the SLIC method, PCR fragments and linearized plasmid were digested by T4 DNA polymerase exonuclease activity for 45 min at 22°C to generate 3′-single-stranded extremities, then all amplification products were mixed in a 1:1:1 molecular ratio and incubated 30 min at 37°C prior to transformation into *E. coli* XL1-blue. The presence of the correct inserts was tested by PCR amplification on white colonies using primers pBSF2 and pBSR3. The sequence of one selected plasmid, pRL55, was further confirmed by sequencing on both strands. Then pRL55 was used to transform strain H26 (Δ*pyrE2*) using the pop-in/pop-out method as described previously. Pop-out colonies were plated on Hv-Ca plates containing 5-FOA and thymidine. The absence of the *hts* and *hdrB* genes was tested by colony lift on 100 colonies from the pop-out plates using a probe targeting *hdrB*. The digoxigenin (DIG)-labeled probe was generated by PCR on H26 genomic DNA using primers RL52 and RL53 and the PCR DIG labeling Mix from Roche. Probe hybridization was detected using the DIG Luminescent Detection kit (Roche) and a ChemiDoc MP (BioRad). Thirty three colonies out of one hundred were deleted for *hts hdrB* genes. Thymidine auxotrophy was confirmed by plating Δ*hts* Δ*hdrB* constructs on both YPC plates and YPC plates containing 165 μM thymidine: colonies could be observed only on YPC plates containing thymidine. One Δ*hts* Δ*hdrB* constructed was conserved for further studies, and designated HvYC0.

### UV irradiation and post-UV recovery assays

For UV (254 nm) irradiation assays on plated cells, cultures were grown to ≈10^8^ cells/ml, diluted in 18% SW and 20 μl aliquots spotted on YPC plates. Once spots were dried, cells were exposed to UV radiation and incubated at 45°C for 4–5 days in the dark for determining the number of survivors. For post-UV recovery assays using cells in liquid media, exponentially growing cells (OD ∼ 0.1) were centrifuged and resuspended in an equal volume of 18% SW. Aliquots were exposed to 50 or 100 J/m^2^ or were not irradiated (control). Cells were centrifuged again, resuspended in an equal volume of *Hv*-YPC broth and grown in the dark at 45°C with agitation prior to imaging.

### Monitoring nascent DNA labeling in Δ*hts* Δ*hdrB* strain

Precultures of *H.volcanii* Δ*hts* Δ*hdrB* (HvYC0) were performed in YPC medium containing 165 μM thymidine. Cells were washed by centrifugation at 4000 rpm for 10 min followed by resuspension of cells to reach OD ≈ 0.1 in fresh YPC medium supplemented with 50 or 100 μM BrdU or 165 μM thymidine. Cell growth was monitored by measuring the OD at 600 nm (Supplementary Figure S1A). BrdU incorporation into DNA was investigated by genomic DNA extraction and dot blot experiments. Genomic DNA (gDNA) was extracted using the Nucleospin Tissue kit from Macherey-Nagel. For each sample, 200 ng of gDNA was spotted on a Nylon membrane Hybond-N (Amersham). gDNA was denatured by incubation in denaturing solution [1.5 M NaCl; 0.5 M NaOH] for 5 min and neutralized by incubation in neutralization solution [1.5 M NaCl; 0.5M Tris–HCl pH 7.5; 1 mM EDTA] for 3 min twice. Then DNA was cross-linked by UV exposure to 0.120 J/cm² (1200 J/m^2^) using a BIO-LINK cross-linker (Vilbert Lourmat, Collégien, France). BrdU detection was performed using a 1/200 dilution of Alexa488-labelled anti-BrdU antibody (Milli-Mark, Darmstadt, Germany) following manufacturer's recommendations. Fluorescence signal was detected using the ChemiDoc MP (BioRad, Marnes-la-Coquette, France). A representative dot blot and its quantification are shown in Supplementary Figure S1B and C, respectively.

### Wide-field fluorescence microscopy of GFP fusion proteins

Cells were mounted on glass slides covered with a thin layer of 1% agarose prepared in 18% SW. Differential interference contrast (DIC, also known as Nomarski interference contrast) and fluorescence images were obtained at room temperature using a ZEISS (Leipzig, Germany) Axio Observer equipped with a 40×, 1.4 NA oil immersion objective. For fluorescence imaging of GFP, 470 nm excitation at a maximum available intensity (5 W cm^−2^) and filter set 65 HE (EX BP 475/30, BS FT 495, EM BP550/100) were used. For each experiment, at least three different *z*-stacks of 30 slices centered on DIC focus (Δ*z* = 0.250 μm) were acquired. For each *z*-stack, we extracted a 2D projection of the maximum intensity using Fiji software (30). Quantitative image analyses were performed on the 2D projections using Fiji. First, cells were segmented by adjusting Otsu's threshold manually. The following items were automatically excluded: (i) objects on the border of the image ‘*Outlines display exclude*’ and ( ii) objects consisting of less than 40 pixels. Then, the GFP signal from the 2D projection was measured in each cell [region of interest (ROI)] to obtain the area of the cell (μm²), and the mean, minimum as well as maximum intensities in arbitrary units (A.U.). The identification of foci was achieved by identifying maxima using the ‘find maxima’ function that determines the local maxima in each cell (ROI) of the image. For these experiments, a noise tolerance of 35 was used, resulting in an output with foci counts in each cell (ROI). For each experiment, at least three z-stacks were acquired and results corresponding to the average of three independent biological replicates were obtained. Unpaired *t*-tests with Welch's correction were performed using GraphPad Prism 5 software. Where indicated, discrete distributions of the foci number were fitted using a Poisson function implemented in the Origin 8 software package using the average of the distribution as the only fitting parameter.

### Localization of nascent BrdU-labeled DNA in Δ*hts* Δ*hdrB* cells

Precultures of *H. volcanii* Δ*hts* Δ*hdrB* (HvYC0) were performed in YPC medium containing 165 μM thymidine. Cells were washed by centrifugation at 4000 rpm for 10 min followed by resuspension of cells to reach OD = 0.25 in fresh YPC medium supplemented with 100 μM BrdU. Cells were grown at 45°C and aliquots were analyzed at various time points. Samples were centrifuged 10 min at 4000 rpm, cell pellets were resuspended in 5 ml 18% SW, where 10 mM Tris–HCl pH 7.5 was replaced by 10 mM HEPES pH 7.5 (HEPES-18SW) containing 4% formaldehyde and incubated 2 h at RT with smooth agitation. Then, fixed cells were washed and resuspended using 60% ethanol containing 3% SW. Cells were washed twice in 18% SW and incubated for one hour at 45°C in a solution containing 35% formamide; 13.5% SW, followed by three washes using phosphate buffered saline (PBS). Immunodetection of BrdU was performed by incubating the cells with anti-BrdU primary antibody coupled with Alexa 488, using a 1/100 dilution (Milli-Mark, ref. 740952), for one hour (Supplementary Figure S1). Cells were washed three times in PBS and resuspended in 18% SW for imaging. Cells were imaged as previously described and the Alexa 488 signal was detected and analyzed using the parameters applied for GFP.

### Wide-field microscopy to study DNA localization and compaction

For DNA labeling by a fluorescent Hoechst 33258 dye (excitation 352 nm/emission 461 nm), cells were centrifuged and resuspended in an equal volume of 18% SW containing 5 μg*/*ml of Hoechst 33258. After 10 min incubation at room temperature in the dark, cells were centrifuged and resuspended in an equal volume of 18% SW. Cells were mounted on glass slides covered with a thin layer of 1% agarose in 18% SW. DIC and fluorescence images were obtained at room temperature using a ZEISS Axio Observer microscope equipped with a 40×, 1.4 NA oil immersion objective. For fluorescence imaging of the GFP signal, 470 nm excitation at a maximum available intensity (5 W.cm^−2^) and filter set 65 HE (EX BP 475/30, BS FT 495, EM BP550/100) were used, whereas 365 nm excitation at a maximum available intensity (2 W.cm^−2^) and filter set 49 (EX G 365, BS FT 395, EM BP 445/50) were used for fluorescence imaging of the Hoechst signal. A *z*-stack (30 slices) centered on DIC focus was performed and data was collected sequentially for Hoechst and GFP signals at each plane. The optical slice with the maximum number of GFP foci was chosen for further study and the Hoechst fluorescence signal was analyzed to determine the mean, minimum and maximum intensities in A.U.

### 3D-Structured Illumination microscopy (3D-SIM)

Cells were mounted on glass slides covered with a thin layer of 1% agarose prepared in 18% SW. Images were acquired on a DeltaVision OMX SR imaging system (OMX version 4.3.9379-1) from GE Healthcare (Velizy, France) with an Olympus PlanApo N 60× 1.42 NA oil immersion objective, using the Cargille labs immersion oil providing an *n*_D_ (at 25°C and 589.3 nm) = 1.5140 ± 0.0002 to minimize spherical aberrations. Imaging was performed using excitation at 488 nm during 25 ms at 40%T (13.2 mW). For each cell, images of 256 × 256 pixels (pixel size of 80 nm) were acquired with a sample thickness of 1 μm (9 sections, Δ*z* = 125 nm) centered on the focus point. At each *z* position, SI images (five phases at three angles) were acquired for 3D-SIM imaging. The quality of the raw SI data was checked by the *SIMcheck* suite of plugins available for Fiji software prior to SI reconstruction performed with the softWoRx software from Applied Precision (Supplementary Figure S2). To highlight the resolution improvement gained using 3D-SIM, an image equivalent to conventional wide-field illumination was generated from raw SI images using the raw SI data to pseudo-wide-field (PWF) utility of the SIMcheck plugin. PWF images were obtained, and quantitative image analyses were performed on both SI and PWF images as described previously: ROI corresponding to cells are defined by manually adjusting Otsu's threshold, and the identification of foci was achieved by identifying maxima with a noise tolerance of 400 and an output style of foci counts in each ROI (cell). The surface area of more than one hundred automatically detected RF were measured by manually limiting their edges. From the obtained area (*A*), the diameter (*D*) of the analyzed foci was calculated using the formula: *D* = *2*√(*A*/π).

### FRAP experiments

The samples were prepared as described for 3D-SIM imaging, and FRAP experiments were also performed on the DeltaVision OMX SR imaging system from GE Healthcare previously described. A spot size of approximately 250 nm was selected and bleached using the 405 nm channel with a 3%T (0.66 mW) excitation for a total bleach duration of 50 ms. In total, 101 wide-field images were collected including ten pre-bleach frames at 500 ms/frame for a total acquisition time of 50 s. Background intensity trace *I*_b_(*t*) was obtained from an ROI outside the cell, and the bleaching of the sample during the experiment *I*_sb_(*t*) was obtained from an ROI inside the cell excluding the photobleached region. The raw fluorescence intensity of the bleached area I(t) was normalized using ${{\rm{I}}_{\rm{n}}}\ ( {\rm{t}} ) = {\rm{\ }}[ {{\rm{I}}( {\rm{t}} ) - {{\rm{I}}_{\rm{b}}}( {\rm{t}} )} ]/{{\rm{I}}_{{\rm{sb}}}}( {\rm{t}} )$. A mono-exponential function was used to fit each normalized trace from different experiments (11 for control cells, 20 for APD-treated cells and 14 for phleomycin-treated cells) using the following formula

\begin{equation*}A - B \times \ {\rm{exp}}\left( { - k \times t} \right)\end{equation*}

where *A, B* and *k* are fitting parameters. *A* and *B* are related to the mobile and immobile fractions of fluorophores: $immobile\ fraction\ = \ 1 - a$ and $\ mobile\ fraction\ = \ b$. *k* (s^−1^) is the recovery rate of the bleached area which was directly converted into the recovery half-time τ_1/2_ (s) calculated as τ_1/2_ = ln(2)/*k*. Unpaired *t*-tests with Welch's correction were performed using GraphPad Prism 5 software.

## RESULTS

### *H. volcanii* RPA2 protein as a proxy for haloarchaeal DNA replication and repair

As a first approach to label *H. volcanii* replication and repair complexes in living cells, we considered the use of GFP-PCNA fusions as replisome markers. However, as stated in the material and methods section, we were unable to replace the chromosomal PCNA gene with the DNA constructs carrying GFP either at the amino- or carboxy-terminal regions of PCNA (HVO_0175), possibly due to steric hindrance of interactions of the replication clamp with other essential replication proteins. Consequently, we decided to create GFP fusions with halophilic single-stranded DNA binding proteins. *H. volcanii* encodes three RPA homologs, named RPA1 (HVO_1338), RPA2 (HVO_0519), and RPA3 (HVO_0292) (31,32). Note that these same proteins are also referred to as RpaA, RpaC, and RpaB, respectively (33). Previous genetic studies revealed that cells lacking RPA1 (and its associated proteins) or RPA3 (and its associated protein) are viable, but that obtaining a double mutant lacking both RPA1 and RPA3 genes was not possible (32,33), strongly suggesting that RPA1 and RPA3 share an essential function. By contrast, RPA2 was found to be essential for viability, as *H. volcanii* cells deleted for a *rpa2* gene cannot be isolated and the down-regulation of *rpa2* expression under the control of a tryptophan-inducible promoter leads to little or no growth in the absence of tryptophan. Moreover, RPA2 overexpression enhances resistance to DNA-damaging agents (33). These observations indicate that RPA2 plays a key role in DNA replication and repair in this halophilic archaeon and justify the use of the *H. volcanii* RPA2 protein as a proxy for archaeal DNA replication and repair.

### Functional expression of the GFP-labeled RPA2 protein in *Haloferax volcanii* cells

**Figure 1.**
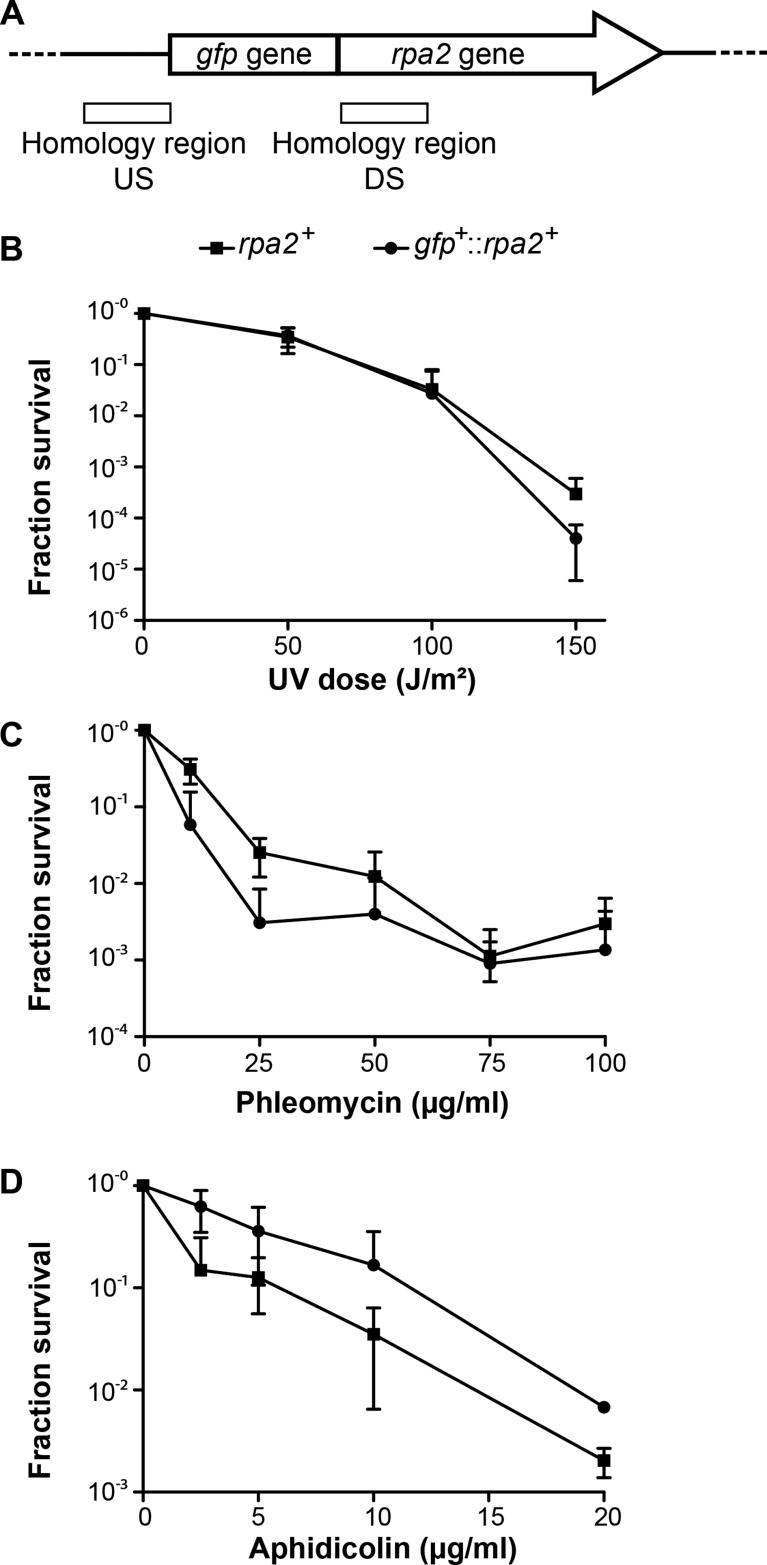
*gfp*-fused *rpa2* allele construction and functional characterization. (**A**) Representation of the chromosomal locus of the *gfp*^+^::*rpa2*^+^ allele. The regions of homology between the plasmid and the chromosome used for pop-in/pop-out gene replacement are represented by white boxes [Up-Stream (US) and Down-Stream (DS) regions]. Surviving fractions for *rpa2^+^* (▪) and *gfp*^+^::*rpa2*^+^ cells (•) in response to (**B**) increasing doses of UV irradiation, (**C**) increasing concentrations of phleomycin and (**D**) increasing concentrations of aphidicolin. Error bars represent standard deviations of at least three independent experiments.

To investigate the *in vivo* localization of DNA replication and repair complexes in *H. volcanii*, we engineered a strain expressing GFP fused to the amino terminus of RPA2 (HVO_0519) from the native chromosomal locus. We first constructed the plasmid pFD2 that carries the upstream region of *rpa2*, the *gfp* gene encoding the smRS-GFP variant that has been successfully used in *H. volcanii* cells (21,34), and the first 400 bp of the *rpa2* gene. The resulting construct carrying an in-frame fusion of *gfp* and *rpa2* was integrated at the *rpa2* chromosomal locus of *H. volcanii* H26 using the pop-in/pop-out method (Figure 1A). All colonies resulting from excision of the plasmid from the chromosome (pop-out) showed comparable growth characteristics on solid media. In 20% (2 out of 10) of the clones tested, the *gfp^+^*::*rpa2^+^* fusion was inserted at the chromosomal *rpa2* locus allowing expression of a GFP-labeled RPA2 protein from its native promoter. One representative clone carrying a chromosomal *gfp*^+^*::rpa2*^+^ (HvFD1) was selected for further study. The generation time of the *gfp*^+^*::rpa2*^+^ strain was not significantly different from the *rpa2^+^* H26 cells [2.2h±0.4 and 1.9h±0.4 respectively (Supplementary Table S2)], establishing that the GFP:RPA2 fusion complements essential functions of RPA2. To further confirm the functionality of this fusion protein, HvFD1 (*gfp*^+^*::rpa2*^+^ ) cells were exposed to DNA damaging agents. The survival of *gfp*^+^*::rpa2*^+^ cells challenged with UV radiation (Figure 1B) was similar to the control cells up to 100 J/m^2^, whereas at 150 J/m^2^ cells carrying a wild type *rpa2* survived better (one log difference). The survival of the *gfp::rpa^2+^* strain in the presence of the DNA-cleaving agent phleomycin appeared ∼5-fold less when compared with *rpa2^+^* cells (Figure 1C), but this difference was close to the observed standard deviations of individual data points. Finally, *gfp*^+^*::rpa2*^+^ cells were also exposed to aphidicolin, which blocks DNA synthesis in halophilic archaea (35). Surprisingly, *gfp*^+^*::rpa2*^+^ cells exposed to increasing doses of aphidicolin were apparently less affected than *rpa2*^+^ cells (Figure 1D). Using Western blot analyses, we did not observe major modulation in a steady-state expression level of the studied GFP fusion protein under the different conditions tested (Supplementary Figure S3). Taken together, these results indicate that the GFP::RPA2 fusion protein fulfills the essential role(s) of RPA2 not only in DNA replication, but also may actively respond to DNA damaging agents and replication inhibitors.

### GFP::RPA2 forms fluorescence foci in exponentially growing *Haloferax volcanii* cells

**Figure 2.**
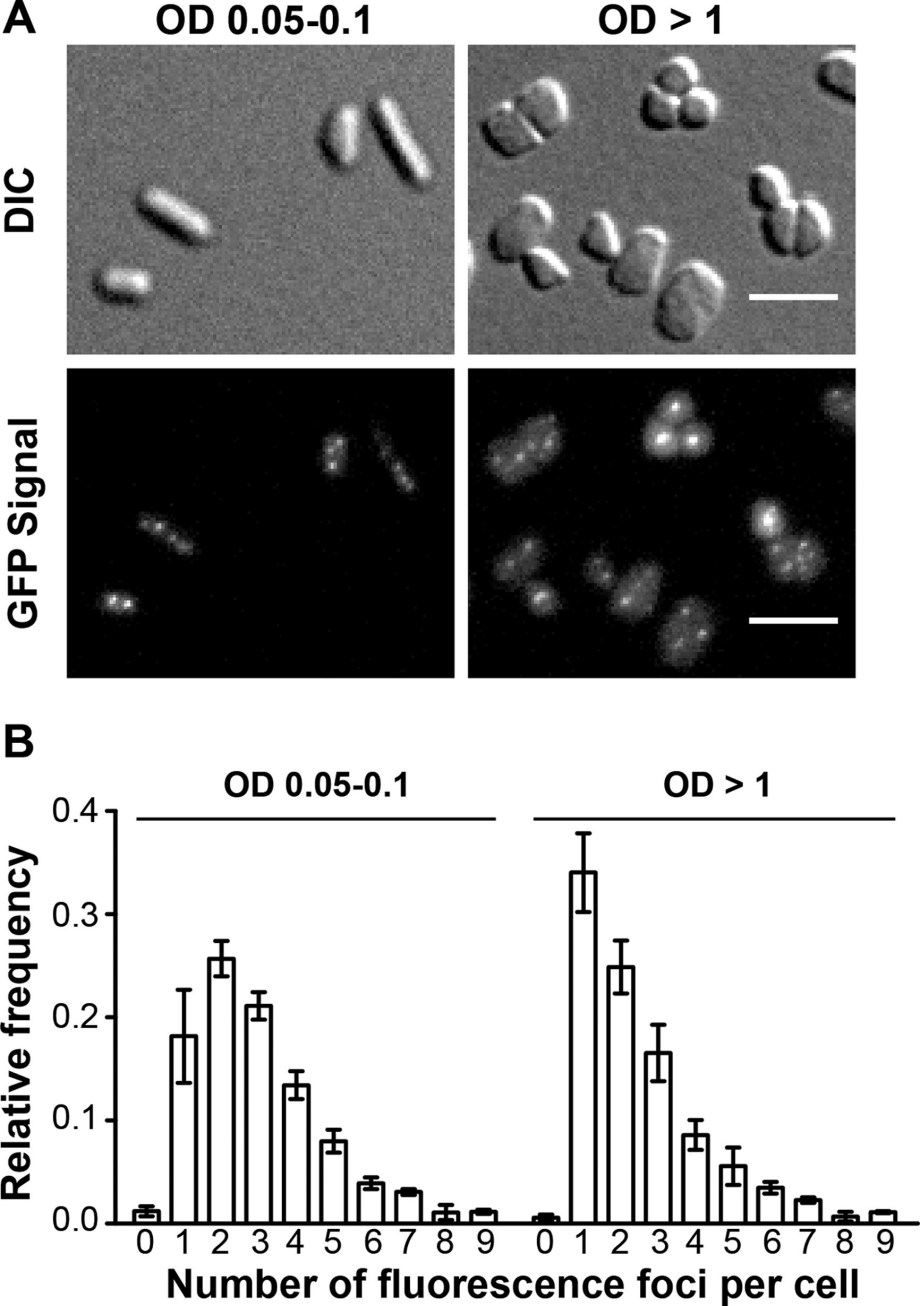
*In vivo* localization of GFP-labeled RPA2 under normal growth condition. A total of 391 cells at OD 0.05–0.1 and 1597 cells at OD >1 were analyzed. (**A**) Images of DIC and GFP signal of *gfp*^+^::*rpa2*^+^ cells at OD 0.05–0.1 and at OD >1 are indicated. A scale bar equals 5 μm. (**B**) Relative frequency of number of foci per individual cell is shown. Error bars represent standard deviations of at least three independent experiments.

We studied the cellular localization of the functional GFP-tagged RPA2 protein in living *H. volcanii* cells under optimal growth conditions using enriched growth medium. For these experiments, *gfp*^+^*::rpa2*^+^ cells were placed on a thin agarose slice, covered with a glass coverslip and subjected to wide-field imaging with an Axio Observer ZEISS microscope using a 40×, 1.4 NA, oil objective (0.26 μm resolution). In exponentially growing *gfp*^+^*::rpa2*^+^ cells [optical densities (OD) ranging from 0.05 to 0.1], we observed well-defined RF (Figure 2A), whereas we observed only a very diffuse fluorescence signal in cells expressing non-fused GFP (21). The average number of fluorescence foci measured in exponentially growing *gfp*^+^*::rpa2*^+^ cells was 3.3 ± 0.6 foci (391 cells analyzed). These GFP::RPA2 foci were also observed in cells reaching stationary phase (OD ≥ 1), but their average number of 2.8 ± 0.3 (1597 cells analyzed) was slightly decreased in comparison to exponentially growing cells (Figures 2A and B). In both cases, the number of RF followed closely a Poisson distribution between one and nine RF, with the fitted average value of 2.76 ± 0.12 (exponentially growing cells) and 2.10 ± 0.20 (stationary phase cells), as shown in Supplementary Figure S4A and B. On the other hand, the number of cells (<2%) with no detectable RF was much less than expected from a Poisson distribution (Supplementary Figure S4). These results thus indicate that rapidly proliferating cells have a slightly increased number of RF when compared to stationary phase cells and revealed significant cell-to-cell variation following a Poisson distribution.

As the RPA2 foci were observed in exponentially growing cultures in the absence of exogenous DNA damaging agents, by analogy to earlier studies, these foci may reflect the *in vivo* localization of active DNA replication forks. However, we do not exclude the possibilities that RPA2 may also associate with spontaneously stalled or collapsed replication forks and/or chromosomal regions undergoing DNA repair in response to spontaneous DNA damage.

### Nascent DNA labeling reveals distinct replication foci in fixed *H. volcanii* cells

**Figure 3.**
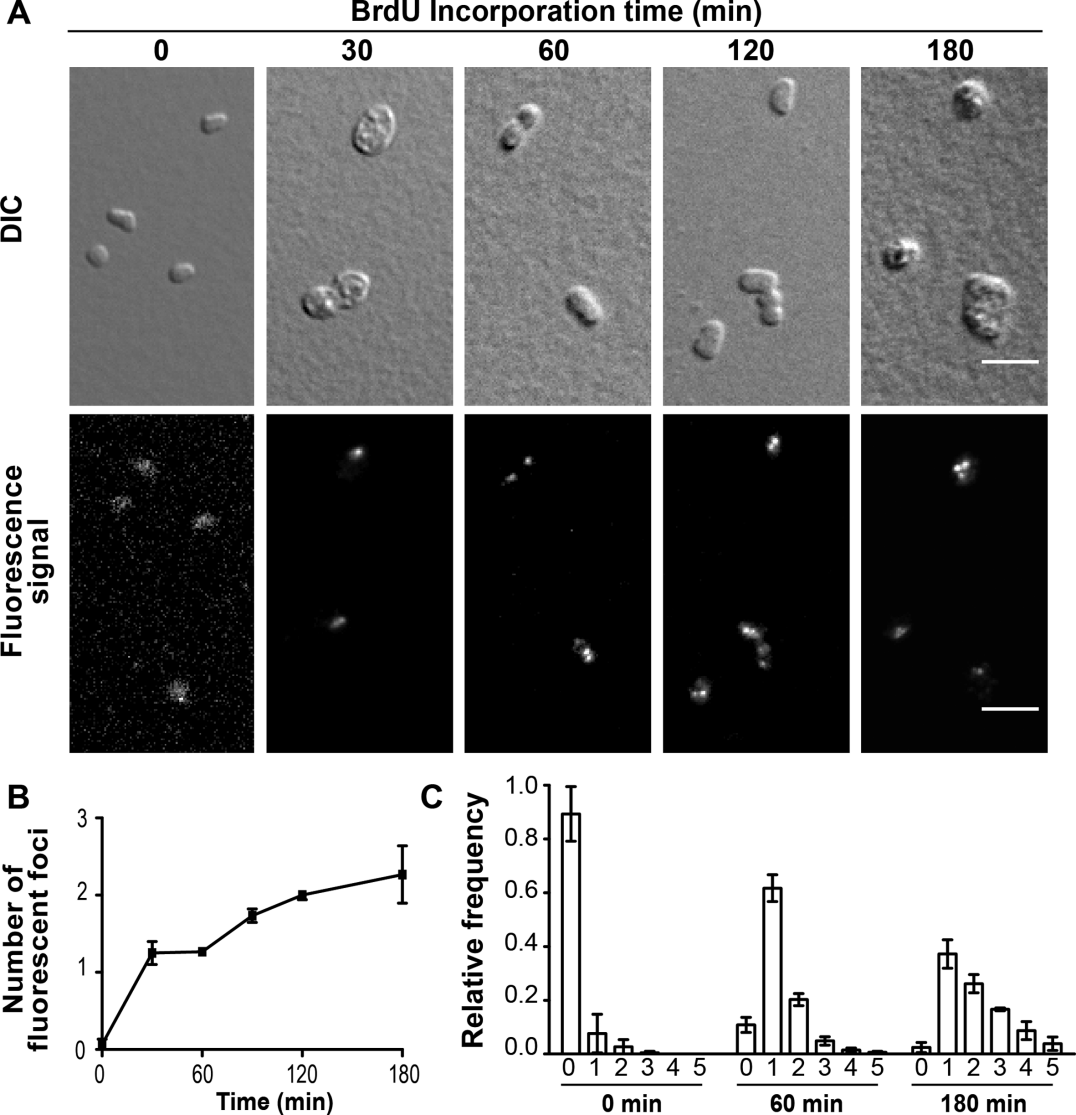
Intracellular localization of nascent DNA. A total number of 163 cells were analyzed at the starting time point of BrdU incorporation, 139 after 30 min, 281 after 60 min, 951 after 120 min and 260 after 180 min. (**A**) DIC images and Alexa 488 signal of anti-BrdU antibody of Δ*hts* Δ*hdrB* cells grown in rich media containing 100 μM BrdU. A scale bar equals 5 μm. (**B**) Mean number of nascent DNA foci formed observed using BrdU labeling and immunodetection of fixed cells. (**C**) Relative frequency of number of nascent DNA foci per individual cell. Error bars represent standard deviations of at least three independent experiments.

To investigate the intracellular localization of nascent DNA synthesis in *H. volcanii*, we engineered a thymidine auxotrophic strain by deleting both *hts* and *hdrB* genes encoding thymidylate synthase and dihydrofolate reductase, respectively. Both genes are involved in thymidylate synthesis (36) and form an operon on the *H. volcanii* chromosome, facilitating their simultaneous deletion by the pop-in/pop-out method (37). The resulting Δ*hts* Δ*hdrB* strain was auxotrophic for thymidine and could not grow on solid or liquid YPC media in the absence of thymidine. By growing cells in YPC media containing 5-bromo-2′-deoxyuridine (BrdU), we could show that Δ*hts* Δ*hdrB* cells incorporate thymidine analogs into nascent DNA more efficiently than wild-type cells (Supplementary Figure S1). The intracellular localization of nascent DNA was investigated by immuno-localization of BrdU-labeled DNA using Alexa 488-labeled anti-bromodeoxyuridine antibodies after cell fixation, permeabilization of cell membranes and DNA denaturation. Discrete fluorescence foci were detected in exponentially growing cells after 30 min incubation in the presence of BrdU. Their average number *per* cell increased from 1.3 ± 0.1 (60 min of BrdU labeling) to 2.3 ± 0.6 (180 min) (Figure 3A and B). Under these conditions, the vast majority of cells contained one to five nascent DNA foci (>90%), whereas <10 % of the cells were not undergoing replication at all, as witnessed by the small number of cells without detectable BrdU foci (Figure 3C). These results thus indicate that in non-synchronized exponentially growing cultures the vast majority of the individual *H. volcanii* cells are engaged in DNA synthesis, as suggested by formation of RF as described above.

### UV irradiation decreases GFP::RPA2 foci number

**Figure 4.**
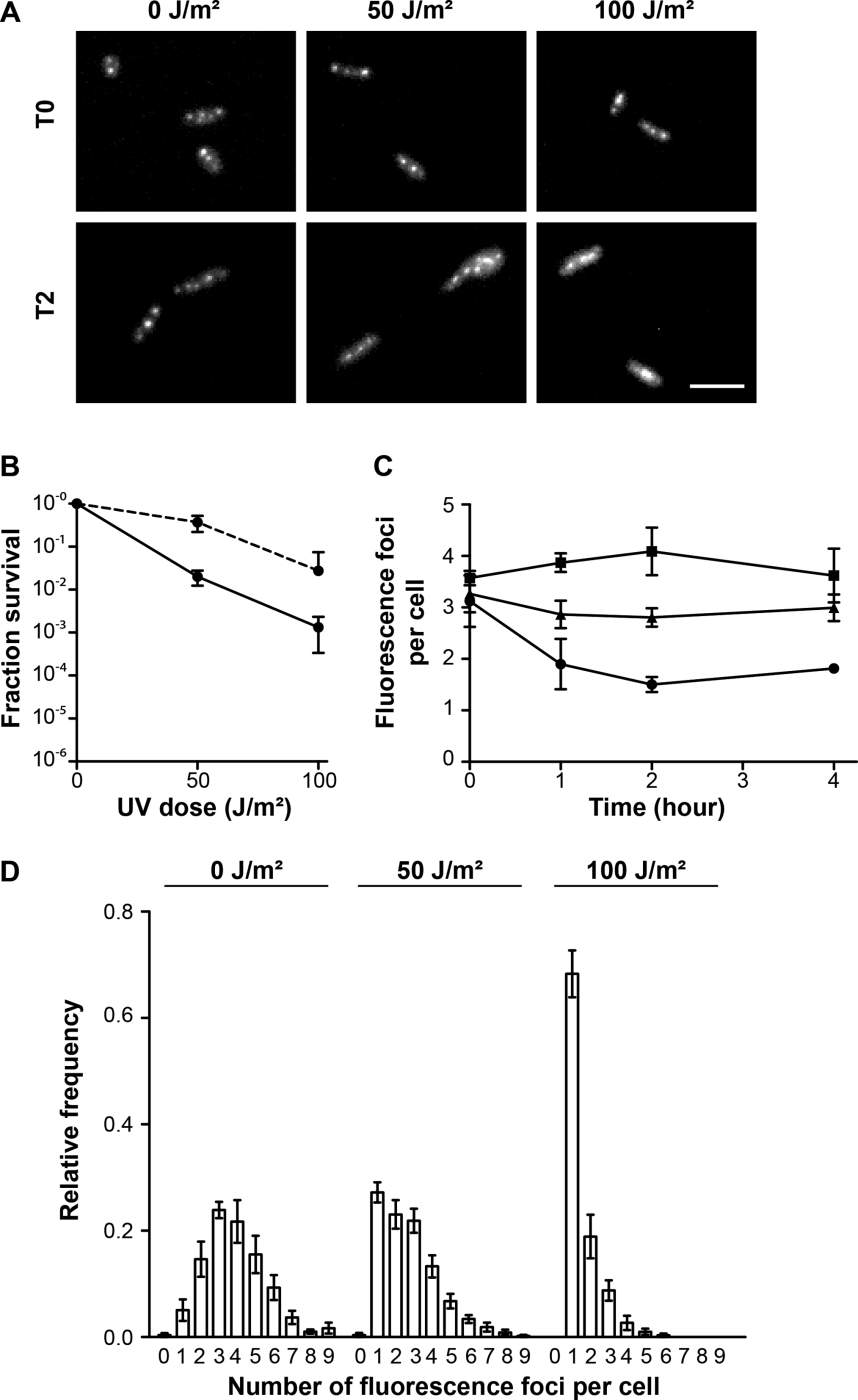
*In vivo* localization of GFP-labeled RPA2 after UV irradiation. A total of 391 cells at OD 0.05–0.1 and 1597 cells at OD >1 were analyzed. (**A**) Images of GFP signal of *gfp*^+^::*rpa2*^+^ cells unirradiated (0 J/m²) or after 50 and 100 J/m² UV irradiation, without recovery (T0) and after two hours recovery (T2). A scale bar is equal to 5 μm. (**B**) Surviving fractions of *gfp*^+^::*rpa2*^+^ cells in response to increasing doses of UV irradiation performed on plated cells (dotted line, as in Figure 1B) and in liquid cultures (continuous line). (**C**) The average number of fluorescence foci *per* cell as a function of time in unirradiated cells (▪) and after 50 (▴) and 100 J/m² (•) UV irradiation. (**D**) Relative frequency of number of foci per individual cell after 2 h recovery. Error bars represent standard deviations of at least three independent experiments.

*H. volcanii* RPA2 is involved in repair of DNA damage triggered by UV irradiation (33), prompting us to investigate GFP::RPA2 intracellular localization in cells treated with UV. Irradiation of exponentially growing cells was performed in liquid medium, followed by a recovery period and imaging. As this recovery phase took place in the dark, DNA repair of UV damaged DNA was dependent on the Nucleotide Excision Repair (NER) pathway. Using liquid cultures, a 10-fold decrease in cell survival was observed upon irradiation of cells with 50 and 100 J/m² compared to plated cells (Figures 4A and B). Because UV irradiation was performed on plated cells having reached stationary phase (OD > 1.0), these results may reflect a higher sensitivity of exponentially growing cells to UV irradiation. In both control cells and cells exposed to 50 J/m² (Figures 4C and D), the average number of GFP::RPA2 fluorescence foci remained constant throughout the experiment and the number of GFP::RPA2 foci in cells treated with 50 J/m² UV irradiation was similar to that observed in UV-treated stationary phase cells. The numbers of RF in the different UV-treated samples were also Poisson distributed with average values of 3.87 ± 0.15 (non-irradiated cultures), 2.50 ± 0.20 (50 J/m^2^) and 1.50 ± 0.40 (100 J/m^2^), as shown in Supplementary Figure S4C. In cells exposed to higher UV doses that decreased cell survival, the average number of GFP::RPA2 foci dropped significantly during the first two hours and then remained stable for 2 h (Figure 4B), indicating that maximum effect of UV irradiation was observed. Under these conditions, the cells with a single focus represented only 5 ± 4% (no UV) in control cells, but their number increased in a dose-dependent manner. The UV-induced single focus was relatively large, particularly in cells treated with 100 J/m^2^, as illustrated in Figures 4A and D. Overall, these results suggested that GFP::RPA2 foci dynamically respond to UV-induced DNA damage, frequently resulting in the formation of a single large focus at high doses of UV radiation.

### Replication stress and DNA breaks result in formation of distinct RPA2 complexes

**Figure 5.**
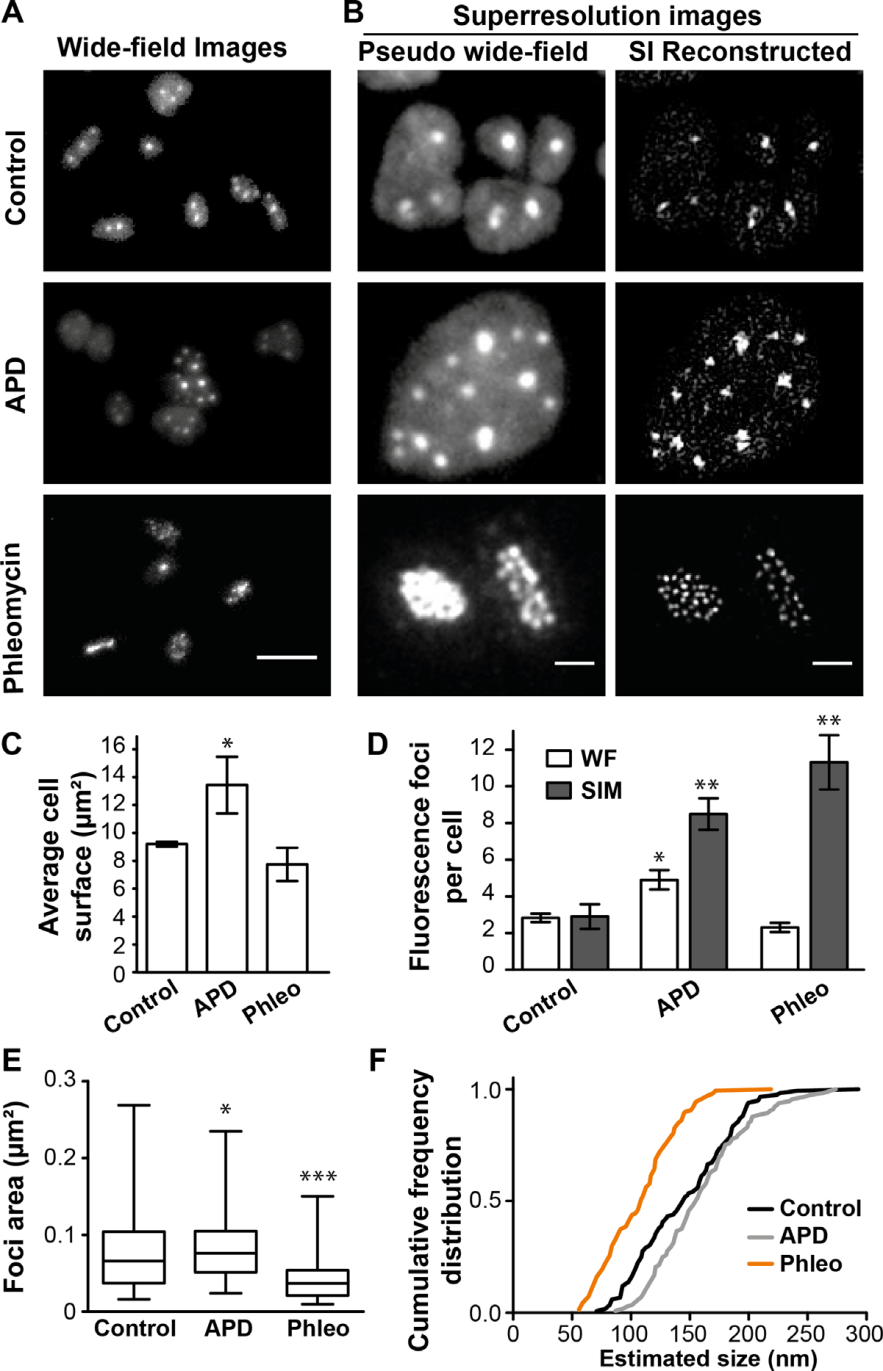
*In vivo* localization of GFP-labeled RPA2 using SIM microscopy. A total of 144 control cells, 75 cells exposed to 5 μg/ml aphidicolin, and 116 cells exposed to 100 μg/ml phleomycin, were analyzed. (**A**) Images of GFP signal of *gfp*^+^::*rpa2*^+^ cells using wide-field microscopy. Bar equals 5 μm. (**B**) Images for each condition of GFP signal of *gfp*^+^::*rpa2*^+^ cells using SIM microscopy, comparing pseudo-wide field (PWF) image with the image obtained after SIM reconstruction. Bar equals 1 μm. (**C**) Average cell surface. (**D**) Mean number of GFP-RPA2 labeled fluorescence foci obtained when analyzing PWF images (light histograms) and SIM images (grey histograms). (**E**) Foci area measured on images after SIM reconstruction. A total of 116 foci were analyzed for control cells, 112 for cells exposed to aphidicolin and 140 for cells exposed to phleomycin. (**F**) Cumulative frequency distribution of the foci size estimates approximated as circles. Error bars represent standard deviations of at least three independent experiments. Unpaired *t*-test with Welch's correction were performed in comparison to control cells. ***Significantly different, *P* < 0.001; **Significantly different, *P* < 0.01; *Significantly different, *P* < 0.05.

Next, we investigated the effect of the DNA synthesis inhibitor aphidicolin on the intracellular localization of GFP::RPA2 fluorescence foci. Using diffraction limited microscopy, exposure to 5 μg/ml of aphidicolin significantly increased the average number of GFP::RPA2 fluorescence foci from 2.8 ± 0.3 (in the absence of the drug) to 4.9 ± 1.0 foci *per* cell with a concomitant increase in cell size (Figures 5A and B), in agreement with our earlier observations (21). We also used the super-resolution 3D-SIM (Structured Illumination Microscopy) technique (38), which improves the lateral resolution to ≈100 nm, to study aphidicolin treated cells. Our 3D-SIM experiments indicated that aphidicolin markedly increased the foci number when compared to untreated cells (Figure 5C and D). Owing to the improved lateral resolution, 3D-SIM revealed a subset of closely located individual foci in SI reconstructed images that were not fully resolved in wide-field or pseudo wide-field images.

We also exposed *H. volcanii* cells to phleomycin, which creates double-strand and/or single-strand breaks with poorly defined DNA extremities in this species (39). In the majority of phleomycin-treated cells (100 μg/ml), using diffraction-limited microscopy, we observed a single fluorescence focus that appeared significantly larger than the RF observed in untreated or aphidicolin-treated cells (Figure 5A). The use of 3D-SIM microscopy, however, clearly revealed multiple discrete fluorescence foci (Figures 5B and D). Foci quantification using 3D-SIM images revealed that phleomycin increased the average number of GFP::RPA2 fluorescence foci and also changed the intracellular localization of RPA2 fusion proteins, when compared to untreated cells (Figure 5).

We further exploited our 3D-SIM data by estimating the diameter of the observed RPA2 foci. First, the surface areas of more than one hundred RF corresponding to the different experimental conditions were determined as described in the Materials and Methods section. The obtained mean surface areas ± standard deviation for DMSO (control), aphidicolin and phleomycin treated cells were 0.073 ± 0.041 (*n* = 116), 0.086 ± 0.046 (*n* = 112) and 0.0399 ± 0.023 μm^2^ (*n* = 137), respectively, which typically represented far <1% of the total cell surface area (Figure 5C). By approximating these surface areas as circular foci, we obtained a cumulative distribution for the diameter of each studied object (Figure 5F). The estimated values ranged from 50 to 300 nm, indicating considerable heterogeneity in the architecture of the detected RPA2 complexes. The mean values of the determined size estimates were approximately 150 nm for control [95% confidence interval (95% CI): 139–154 nm] and aphidicolin (95% CI: 153–168 nm) treated cells. However, the foci brought about by phleomycin treatment appeared systematically smaller with an estimated mean diameter of 108 nm (95% CI: 103–113 nm).

These results indicate that the different conditions and treatments used here may result in the formation of varying amounts of exposed ssDNA bound by different numbers of RPA2 molecules in live *H. volcanii* cells.

### Phleomycin-triggered formation of RPA2 foci coincides with DNA compaction

**Figure 6.**
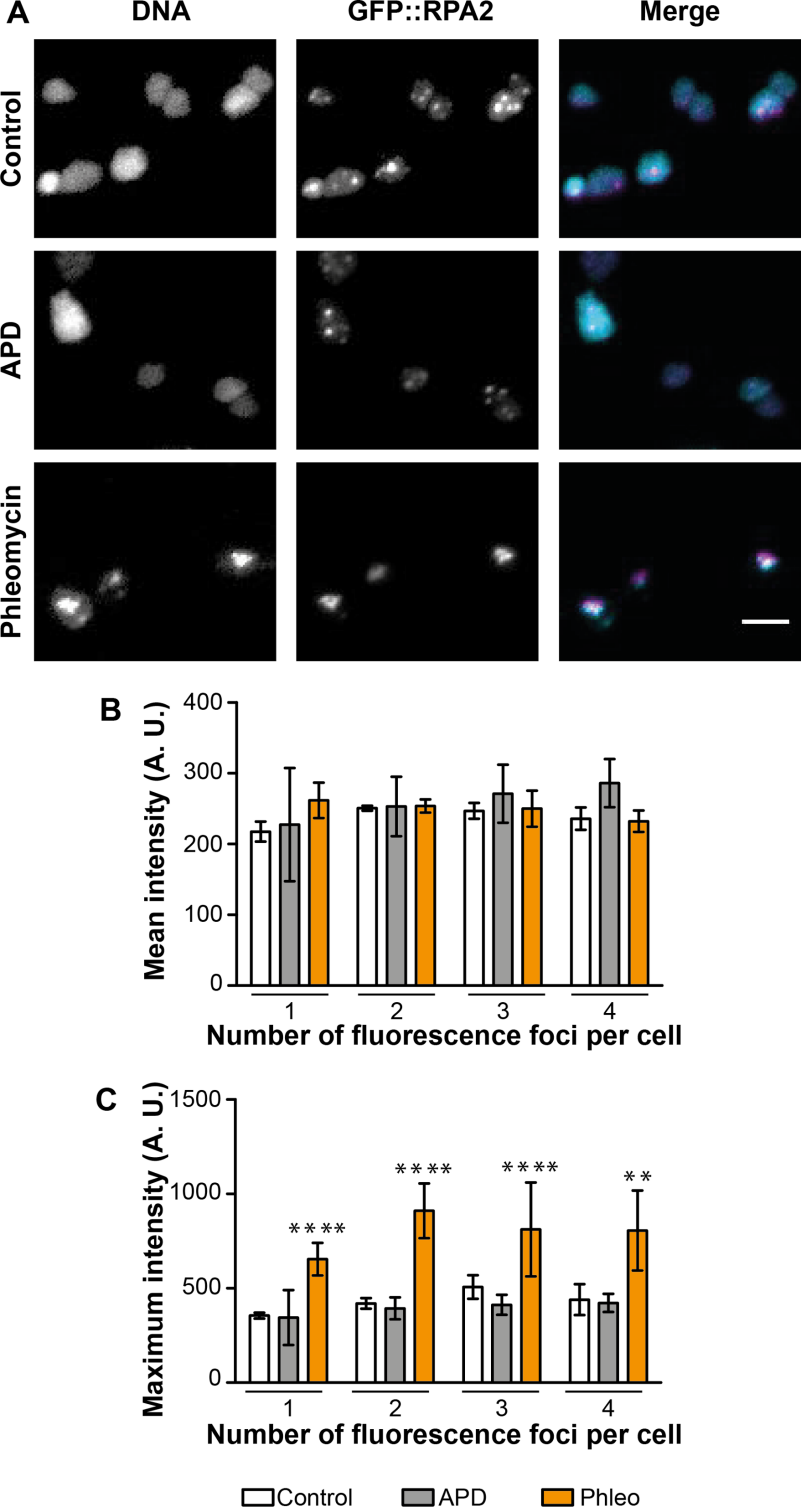
*In vivo* localization of Hoechst-labeled DNA and GFP-labeled RPA2 using wide-field microscopy. A total of 274 control cells, 159 cells exposed to 5 μg/ml aphidicolin, and 389 cells exposed to 100 μg/ml phleomycin, were analyzed. (**A**) Images for each condition of the Hoechst-labeled DNA, the GFP signal and the merge image of the DNA signal in cyan and the GFP signal in magenta. Bar equals 5 μm. (**B**) Mean intensity (A.U.) and (**C**) Maximum intensity (A.U.) of the Hoechst-labeled DNA signal in cells depending on the individual number of GFP::RPA2 foci. Light histograms correspond to control cells, grey ones to aphidicolin-treated cells and orange ones to phleomycin-treated cells. Error bars represent standard deviations of at least three independent experiments. Unpaired *t*-test with Welch's correction were performed in comparison to control cells. ****Significantly different, *P* < 0.0001; **Significantly different, *P* < 0.01.

DNA damaging agents induce DNA compaction in a dose-dependent manner in *H. volcanii* (40). Thus, we quantitatively investigated DNA compaction of cells exposed to 5 μg/ml aphidicolin or 100 μg/ml phleomycin by wide-field fluorescence microscopy using a fluorescent DNA binding dye. For each observed cell, the DNA labeling signal (DNA compaction) was determined at the same focal plane where most of the GFP::RPA2 foci were observed. In the majority of control cells and cells exposed to aphidicolin, a rather diffuse cytosolic DNA signal was observed, as shown in Figure 6A. In contrast, in the majority of the phleomycin-treated cells, the DNA signal appeared much brighter and significantly more condensed. We performed quantitative analyses of the DNA signal in the cells containing one to four GFP::RPA2 foci, as they represent the majority of cells. Although the mean intensity of the DNA signal was similar in all cells (Figure 6B), the maximum localized intensity measured in phleomycin-treated cells was significantly higher compared to untreated cells or cells exposed to aphidicolin (Figure 6C). These results revealed that the DNA content of the cells does not significantly fluctuate between the different experiments, but that the addition of phleomycin triggers DNA compaction and formation of the ‘small’ RPA2 foci described above. It should also be noted that in phleomycin-treated cells, RPA2 co-localized with compacted DNA (Figure 6A).

### Diffusion dynamics of GFP::RPA2 proteins is constant upon DNA damage or replication stress

**Figure 7.**
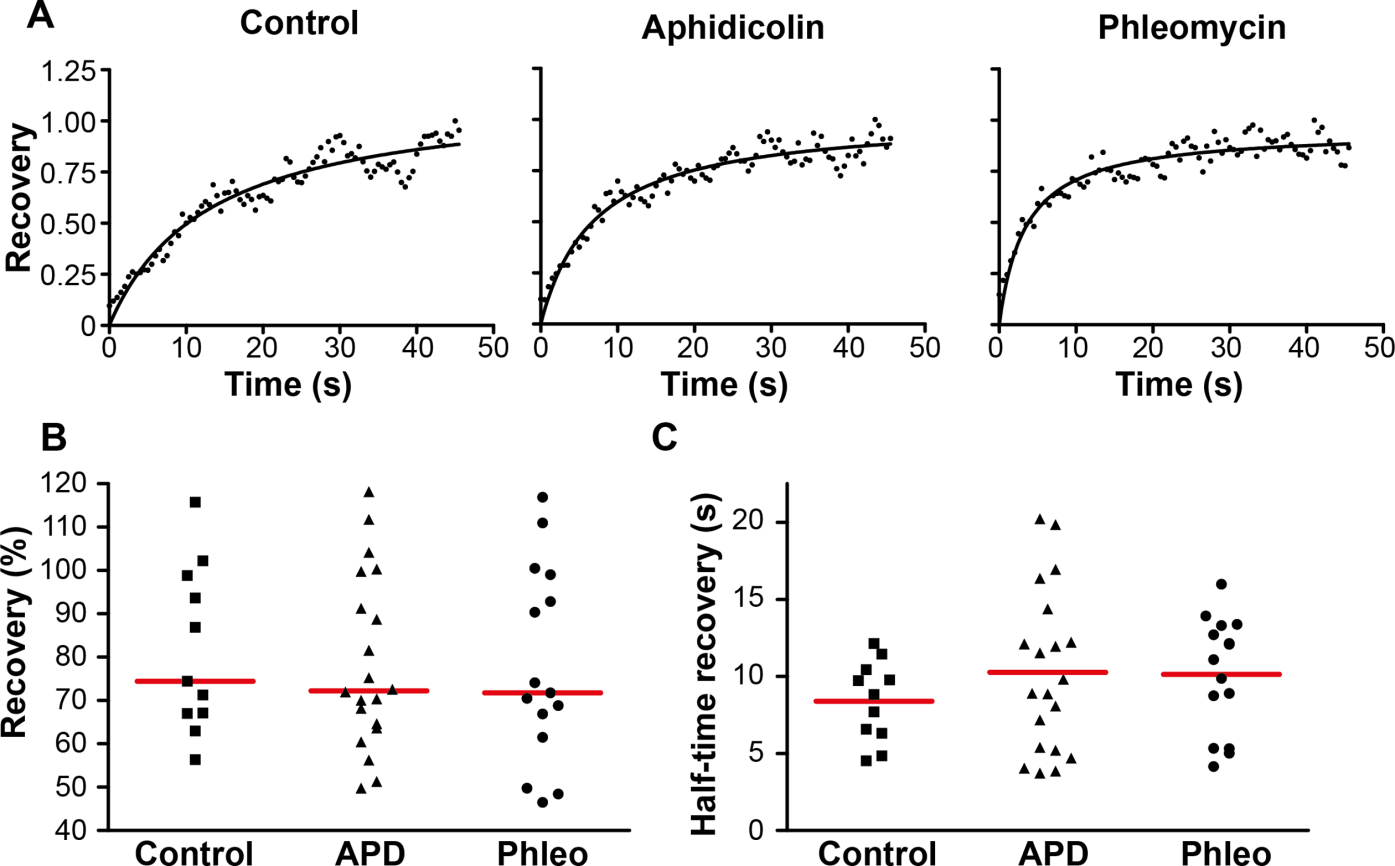
Dynamic localization of GFP-labeled RPA2 molecules at fluorescence foci. A total of 11 control cells, 20 aphidicolin-treated cells and 14 phleomycin-treated cells were analyzed. (**A**) Representative fluorescence recovery measurement (dots) after photobleaching, and the corresponding fitted curve (black line), for each condition. (**B**) Percentage of recovery reached. (**C**) Half-time recovery (s) calculated from the fitted curves. Data for each cell are represented, and the red lines correspond to the means. Unpaired *t*-test with Welch's correction were performed in comparison to control cells and concluded to no significant differences.

To characterize an average mobility of GFP::RPA2 molecules at the foci, we performed fluorescence recovery after photobleaching (FRAP) measurements. For each analyzed cell, a region including one GFP::RPA2 focus was photobleached and fluorescence recovery kinetics were measured using cells exposed to DMSO (control), aphidicolin or phleomycin (Figure 7A). Our results suggested that ≈ 80% of the fluorescence was recovered within twenty seconds in control and drug-treated samples (Figure 7B). In all cases, a mono-exponential fit of recovery curves reflected the existence of one major population of diffusing GFP::RPA2 molecules (Figure 7A). The average recovery half-times, calculated from the fitted curves (Figure 7C), were similar for control cells (8.40 ± 2.6 s) and cells exposed to aphidicolin (10.3 ± 5.2 s) or phleomycin (10.1 ± 3.7 s). These results thus indicate that up to 80% of GFP::RPA2 molecules forming foci are mobile, whereas ∼20% of RPA molecules may remain bound to or associated with DNA during the investigated time span.

## DISCUSSION

We have developed a fluorescent variant of the single-stranded DNA binding protein RPA2 (32,33) and nascent DNA labeling as new tools to study DNA replication and repair in live haloarchaeal cells.

Three RPAs are found in the *H. volcanii* genome (31). We focused on RPA2 as genetic studies had shown that it is essential for cell viability, indicating its central role in replication and repair (32,33). Archaeal RPA/SSB are diverse and display characteristics of both eukaryotic RPA and bacterial SSB, with significant variation in structure, domain organization, and subunit composition. They are nevertheless thought to protect single-stranded intermediates generated during replication, recombination and repair from attack by nucleases and chemical modifications (41–44). Moreover, archaeal RPAs are likely to direct proteins at sites of DNA replication, recombination and repair through direct protein-protein interactions (45–48). Key cellular roles of haloarchaeal RPA proteins have also been suggested by recent genetic studies of the five different RPA homologs in *Halobacterium salinarum* revealing specialized roles depending on the type of DNA damages encountered (49).

We successfully obtained a viable *H. volcanii* strain expressing a GFP::RPA2 fusion from the native chromosomal locus (Figure 1). Our results showed that the GFP::RPA2 fusion protein supported growth, was functional in repair of DNA damages caused by UV irradiation and phleomycin and responded to replication stress caused by the DNA synthesis inhibitor aphidicolin. Combining wide-field imaging and quantitative image analysis, we have demonstrated that GFP-labeled RPA2 proteins form discrete fluorescence foci, referred to as RF, under normal growth conditions (Figure 2). Nascent DNA labeling using a strain engineered during this study revealed that more than 90% of *H. volcanii* cells were actively undergoing DNA replication (Figure 3). These results suggest that a sizeable portion of the observed GFP::RPA2 foci correspond to active replication forks. However, the fact that more RF were detected in living cells than nascent DNA foci in fixed samples may suggest spontaneous accumulation of RPA2 in non-replicative chromosomal regions such as stalled or collapsed replication forks or regions of spontaneous DNA damage. Nevertheless, the *in vivo* localization of RPA2 at active RF was further supported by the dynamic response of RPA2 foci to DNA damages induced by UV irradiation. We observed that the number of the GFP::RPA2 foci decreased in cells containing sufficient DNA damage to block replication and impair cell viability. Moreover, the decrease in cell survival induced by UV irradiation correlated with an increased proportion of cells containing a large single GFP::RPA2 focus (Figure 4). *A priori*, this dynamic response could reflect either the stalling of archaeal DNA replication at pyrimidine dimers or, alternatively, a delayed appearance of replication-induced DNA double-strand breaks formed by replication forks encountering UV-induced single-strand lesions. Note that archaeal replicative DNA primases can bypass cyclobutane pyrimidine dimers (50) and UV-induced replication arrest leading to DNA double-strand breaks has already been demonstrated in Archaea (51). In the case of *H. volcanii*, these UV-induced foci could also reflect the bypass of pyrimidine dimers by translesion DNA synthesis, as the genome of this species contains Family-Y DNA polymerase homologous to the bacterial enzyme DinB (31).

In exponentially growing *Haloferax volcanii*, in the vast majority of cells we observed between one and nine RF using GFP::RPA2 fluorescence (Figure 2) or one to five sites of nascently labeled DNA (Figure 3). The number of the detected RF closely followed a Poisson distribution, whereas the number of cells without foci was less than expected by change (Supplementary Figure S4). As the Poisson distribution is the discrete probability distribution describing the number of independent events occurring in a specific time period, our observations have important biological consequences. In particular, our data imply independent formation of haloarchaeal RF through stochastic processes. This observation is analogous to eukaryotes where spontaneous stochastic firing of individual origins has been proposed (52). Our observations using *H. volcanii* may thus reflect cell-to-cell variability in origin utilization. Alternatively, the heterogeneity of the assembly of replication forks might have been missed in previous studies using cell populations. Note also that our results are very different from what has been previously observed either in bacteria or *Sulfolobus* species mainly carrying two to three RF (7,10,19).

The number of potential active replication forks (more than one hundred without accounting for the two megaplasmids) is much higher than the number of the observed RF. This suggests that the origin firing and assembly of replication complexes could be regulated *e.g*. by ATP-dependent loading of the MCM helicase at origins by the archaeal Orc1/Cdc6 (53,54). Alternatively, the number of active replisomes could be limited by metabolic cues such as phosphate starvation or DNA precursor availability (22). The fact that the number of cells without RF is very small could also indicate that initiation of DNA replication is an efficient, albeit stochastic, process. However, we cannot exclude the possibility of non-replicating cells being non-viable, at least partially explaining their low observed frequency. Differently from *Sulfolobus* (19), we do not observe a clear association of *H. volcanii* RF with the cell periphery, thus making the role of the cytosolic membrane as regulator or organizer of DNA replication less likely. Our work moreover raises the possibility that not all four chromosomal origins of *H. volcanii* H26 are necessarily simultaneously active in exponentially growing single cells. This proposal is consistent with the fact that a single well-defined bidirectional replication origin on the main chromosome supports the growth of genetically modified *H. volcanii* (23) and *H. mediterranea* (55) strains. These marker frequency analyses performed by deep-sequencing or whole-genome microarrays of cell populations have also shown that *Haloferax* replication origins differ in terms of their activation or initiation efficiencies, also supported by studies on copy number control of *H. volcanii* replication origins (56). The progression rate of a single replication fork in euryarchaea is estimated at 15–20 kb/min (57), suggesting that the main chromosome of *Haloferax* could be replicated in 90–115 min from a single replication origin. This estimated replication time from a single replication origin is similar to typical doubling time of *H. volcanii*, thus possibly explaining why multiple origins are not an absolute requirement for cell growth in *Haloferax* species.

We also investigated the effect of the DNA polymerase inhibitor aphidicolin on the intracellular localization and quantity of *H. volcanii* RPA2 foci (Figure 5). In comparison to control cells, inhibition of DNA synthesis increased both the cell size [Figure 5C (21)] and the number of GFP::RPA2 foci (Figure 5D), likely reflecting the formation of stalled replication forks. When 3D-SIM microscopy with a lateral resolution of 100 nm was used for quantification, we observed a three- to four-fold increase in the number of the RPA foci in aphidicolin-treated cells when compared to control cells. Closer inspection of our data revealed that aphidicolin creates larger fluorescence foci in (pseudo) wide-field images frequently resolved as two individual closely located foci in SI reconstructed images. A feasible explanation for these observations is that blocking of DNA synthesis by aphidicolin results in ssDNA accumulation at stalled forks and prevents the physical separation of two closely located replication forks. This observation also raises the possibility that the different replication forks/replicons may be spatially co-localized, providing an alternative explanation for the low number of RF observed in live *H. volcanii* cells, as described above. This is of great interest, as whether all copies of the main chromosome are simultaneously replicated in all cells remains unknown.

We also exploited our GFP::RPA2 fusion to investigate the DNA repair dynamics in *H. volcanii* cells by exposing cells to phleomycin, which triggers DNA strand breaks (Figure 5). This compound changed the intracellular localization of RF when compared to untreated cells (Figure 5A and C). In the presence of phleomycin, a single large focus was observed using wide-field microscopy. 3D-SIM resolved these poorly defined foci as up to ten individual foci *per* cell located within the resolution limit of conventional fluorescence microscopy. We also observed a specific DNA compaction of phleomycin-treated cells and the co-localization of RPA2 with compacted DNA (Figure 6). Such DNA compaction has already been observed in the case of *H. volcanii* (40). This may be part of a DNA damage response that facilitates target search of DNA repair proteins and recognition of intact DNA sequences during homologous recombination. Thus, the specific re-localization of RPA2 in UV-irradiated cells (Figure 4) and phleomycin-treated cells (Figure 5) may reflect this DNA damage response.

Our nanoscopy measurements of archaeal replication and repair structures also provide the first estimates for the diameter of archaeal DNA replication and repair structures in living cells. For instance, we provide a median size estimate of approximately 150 nm, with a range from 80 nm to 250 nm, for RPA2 foci both in control and aphidicolin treated cells (Figures 5E and F). This median value and size heterogeneity are very similar to what has been attributed in earlier studies to the replication domains in human cells using optical super-resolution (16) and electron microscopy (15). In eukaryotes, very recent studies have suggested that on average these replication units of 150 nm correspond to the sites of DNA replication with four co-replicating DNA segments of approximately 20 kb (18,58). As *H. volcanii* replication origins are separated by several hundreds of kilobases (24), we find it unlikely that several clustered *cis*-acting replication origins would be simultaneously processed. Further microscopy studies with improved optical resolution and more efficient nascent DNA labeling (with shorter pulses) are required to further investigate this point. Strikingly, phleomycin-induced repair foci were systematically smaller than RF in control and aphidicolin-treated cells (Figure 5), suggesting that a smaller amount of ssDNA is covered by RPA2. Our FRAP analyses showed that the immobile portion and diffusion properties of RPA2 are very similar under all three conditions (Figure 7), indicating that the biochemical properties of RPA2 molecules are not modulated in the three cases. These *in vivo* observations are fully compatible with earlier *in vitro* studies on archaeal single-stranded DNA binding proteins that have indicated that (hyper)thermophilic or halophilic RPA proteins bind ssDNA with very high affinity in a sequence-independent manner (47,59), but that they also physically interact with other DNA replication, repair and recombination proteins (46).

In conclusion, using wide-field and 3D-SIM super-resolution live cell imaging, as well as nascent DNA labeling, we have provided unexpected insight into the intracellular dynamics of archaeal DNA replication and repair. The small number of the observed RF is unexpected suggesting the presence of regulatory factors of haloarchaeal replication that remain to be identified. Stochastic cell-to-cell variation of RF formation is also of interest to further our still limited understanding on how DNA replication of many replicons found in *H. volcanii* is achieved. We also observe that inhibition of DNA synthesis and chemical agents creating DNA breaks result in the reorganization and/or re-localization of RPA2 complexes. As Archaea and Eukarya share homologous DNA replication and repair proteins, a basic unit of DNA replication may have evolved prior to the divergence of these two domains of life. Altogether our results underline the great potential of live cell imaging to unravel molecular details of fundamental molecular processes in the Third domain of life.

## Supplementary Material

Supplementary Data
