# Supplementary material for: Snapshots of archaeal DNA replication and repair in living cells using super-resolution imaging
Source: Nucleic Acids Res. 2018 Sep 13;46(20):10757–70. doi: 10.1093/nar/gky829 (PMC6237752; doi:10.1093/nar/gky829)
Supplement: Supplementary Data [file gky829_supplemental_files.pdf]

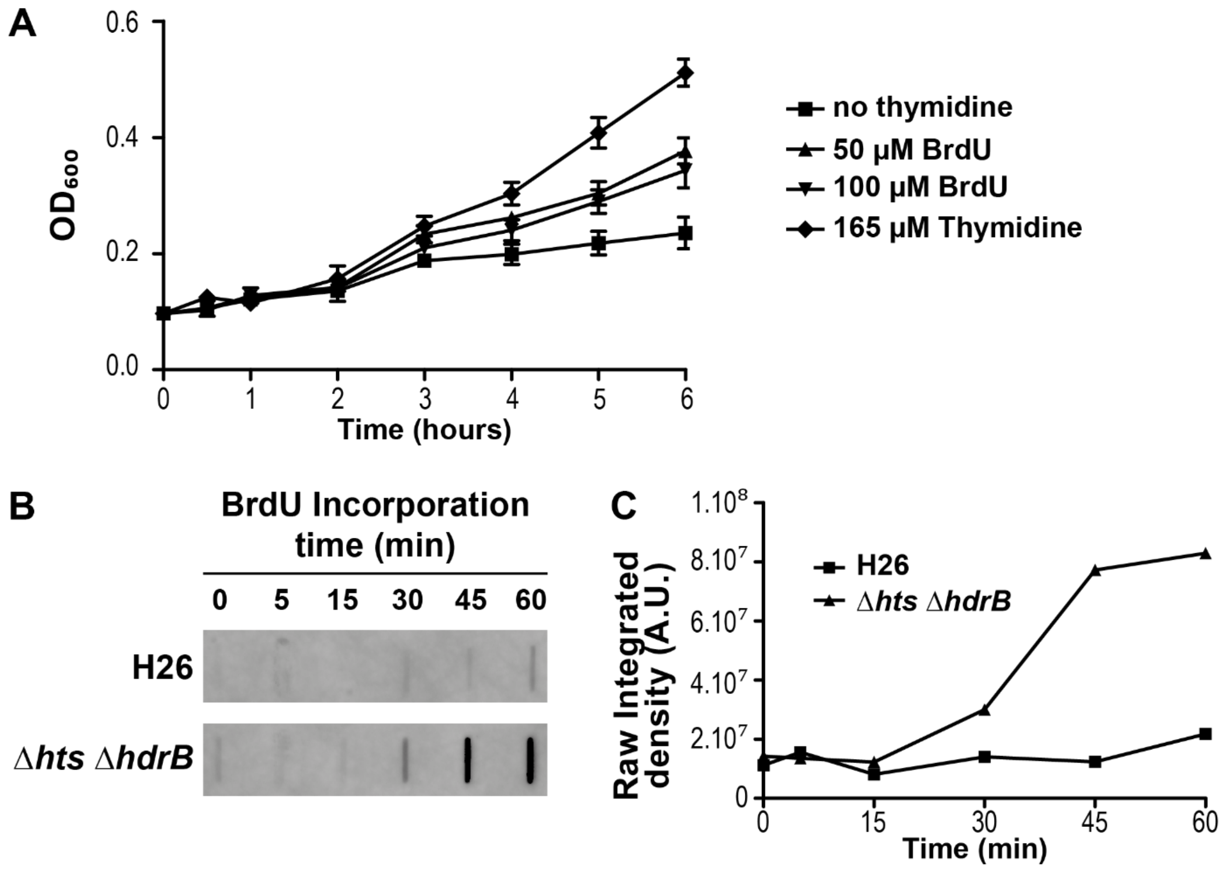

Figure S1: BrdU incorporation in *H.volcanii* Δ*hts* Δ*hdrB* **cells.** (A) Growth curves of Δ*hts* Δ*hdrB* cells in rich media (YPC) (■), or in rich media supplemented with 50 µM BrdU (▲), with 100 µM BrdU (▼) or with 165 µM thymidine (◆). (B) Representative BrdU signal detected by slot-blot analysis of 200ng of genomic DNA after growth in YPC + 100 µM BrdU. H26 cells were compared to Δ*hts* Δ*hdrB* cells. (C) Signal quantification of the signal shown panel B using Fiji. “Substract Background” was performed with a rolling ball radius of 25, then signal was measured for each sample using a single-sized ROI.

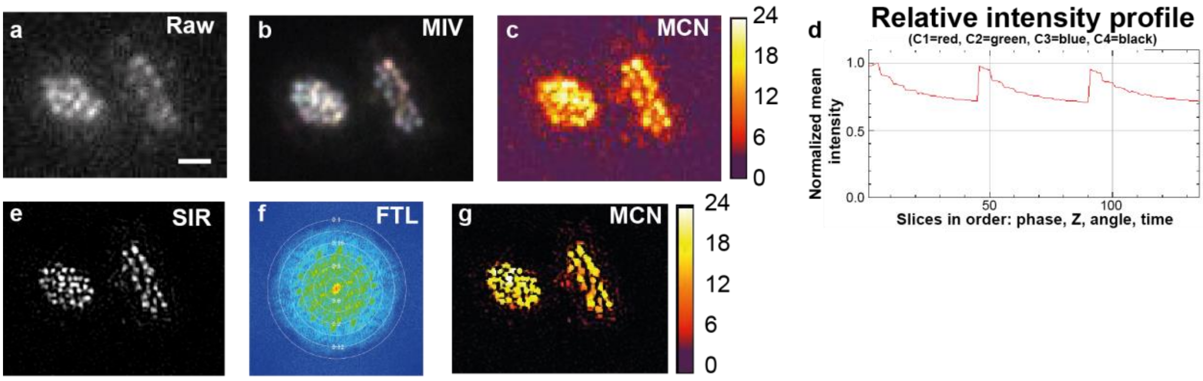

Figure S2: SIMcheck output for raw (a-d) and SIM reconstructed (e-g) data. Scale bar 1.0 µm. (a) Representative image from a raw 3D-SIM data. (b) Output image MIV (Motion and illumination variation) indicating motion stability and evenness of the illumination. (c) Final representation of a heatmap of local modulation of the contrast-to-noise ratio (MCNR) used to optimize the acquisition parameters. (d) Relative intensity profiles according to phase, Z and angle indicating the rate of bleaching and the relative intensity of the illumination pattern for each illumination angle. (e) Representative SIM reconstructed image. (f) Fourier spectra display of lateral FFT illustrating the effective spatial resolution. (g) Output image MCN (Modulation contrast to noise) showing the local variations in reconstruction quality.

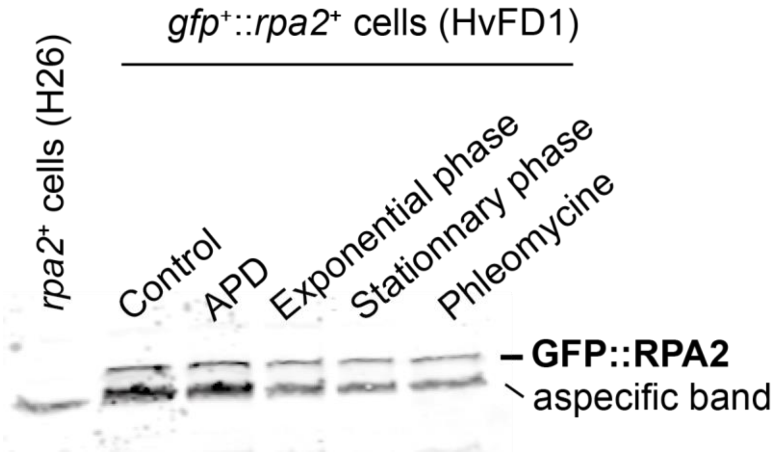

Figure S3: Western-blot to reveal GFP::RPA2 molecules in crude extract using antibodies against GFP. For each condition, lysis of an equal quantity of cells was performed in [2M KCl – 10 mM HEPES – pH 7] by sonication. Lysats were centrifuged 1 hour at 4°C. Proteins from supernatants were separated by electrophoresis in denaturing conditions using NuPAGE 4-12% Bis-Tris Protein Gels (Thermofisher Scientific) following manufacturer instructions. Proteins were transferred on a nitrocellulose membrane and GFP::RPA2 proteins were revealed using a primary antibody against GFP (1/5000) (ORIGENE) and a secondary antibody against rabbit antibodies coupled to IRDye 680 (LICOR). Signal was revealed using a Licor Odyssey Imaging system. A band specific to GFP::RPA2 was revealed with an apparent molecular weight of 120 kDA, higher than the expected molecular weight of 80 kDa, which is often the case with halophilic proteins. Three independent experiments were performed, and results from a representative western blot was shown.

Figures S4A and S4B

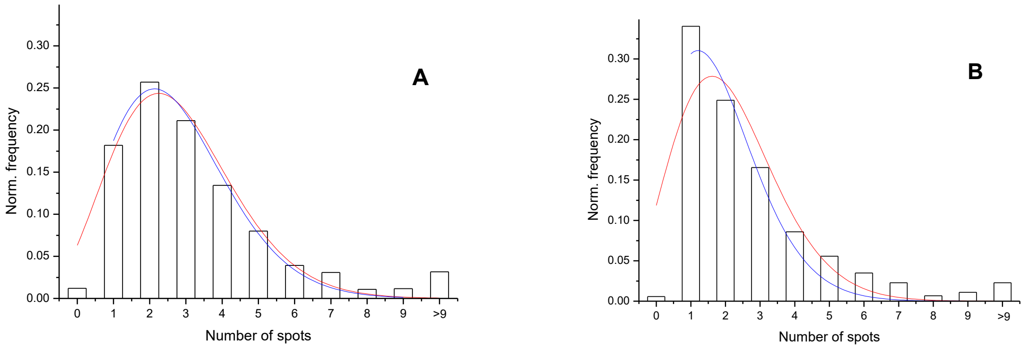

Figures S4A and S4B: The observed number of RF ("spots") fitted using a Poisson function using the average of the distribution as the only fit parameter. In both panels the red plots are fits to points from 0-9 number of spots: for the blue curves the point at 0 number of spots is excluded from the fit. The mean values for the number of spots corresponding to the red and blue curves are 2.76 ± 0.12 and 2.65 ± 0.06 (panel A) and 2.1 ± 0.2 and 1.73 ± 0.12 (panel B). Panels A and B refer to **Figure 2B** of the main manuscript.

Figures S4C

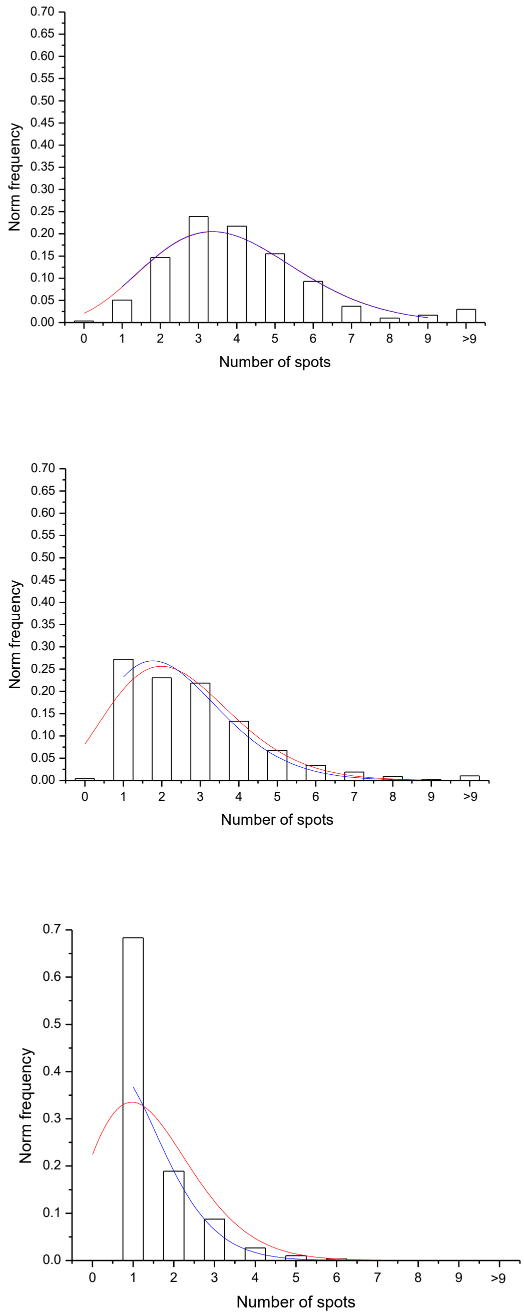

Figures S4C: The fits are to a Poisson distribution with only fit parameter the average of the distribution (referring to **Figure 4D** of the main manuscript). In both panels the red plots are fits to points from 0-9 number of spots: for the blue curves the point at 0 number of spots is excluded from the fit. The mean values for the number of spots corresponding to the red and blue curves are 3.87 $\pm$ 0.15 and 3.86 $\pm$ 0.15 (*upper, no UV treatment*), 2.5 $\pm$ 0.2 and 2.39 $\pm$ 0.11 (*50 J/m^2, middle*) and 1.5 $\pm$ 0.4 and 1.0 $\pm$ 0.5 (*100 J/m^2, lower*).

Supplementary Table S1. Oligonucleotides used during this work.

Primer	Sequence (5' to 3')	Comments/use
**Construction of *H. volcanii gfp*$^{+}$::*rpa2*$^{+}$strain**		
RL129	CGACGCGGAGAACTGCGCC	Amplification of the up-stream region of *rpa2*
RL130	AGTTCCTCGCCTTTCGACATCAGGCGTCACCTCCCGG	Amplification of the up-stream region of *rpa2*, underlined sequence is complementary to *gfp*
RL85bis	ATGTCGAAAGGCGAGGAACTCTTC	Amplification of *gfp*
RL124	CGACCGGTAGGCGTAGCCGC	Amplification of *gfp*
RL131	GCGGCTACGCCTACCGGTCGATGGGCGTCATCCGGGAGG	Amplification of the 5' region of *rpa2*, underlined sequence is complementary to *gfp*
RL132	AGGTTCTCCTTCGCGCCG	Amplification of the 5' region of *rpa2*
BSF2	TTAAGTTGGGTAACGCCAGGG	Screening for fusion construct starting from "white" colonies
BSR3	ACCCCAGGCTTTACACTTTATGC	Screening for fusion construct from "white" colonies
RL141	GCCAGTGTGGACTTCGACCG	Confirmation of the *gfp*$^{+}$::*rpa2*$^{+}$ at the native locus
RL165	GACGGTTACGATGTGAACTCC	Confirmation of the *gfp*$^{+}$::*rpa2*$^{+}$ at the native locus
**Construction of *H. volcanii* Δ*hts* Δ*hdrB* strain**		
RL208	CGACGGTATCGATAAGCTTGATATCGAATTGGCGAGTCGCGACGCTCGGGC	Upstream (US) region of *hts hdrB* operon
RL209	TTTGTACTCCCACTCGCCCGAGTTACTCATGAGAGGTGCCCTGGTTGGGTG	Upstream (US) region of *hts hdrB* operon
RL210	GACGACCAACACCCAACCAGGGCACCTCTCATGAGTAACTCGGGCGAGTGG	Downstream (DS) region of *hts hdrB* operon
RL211	AAAAGCTGGAGCTCCACCGCGGTGGCGGCCACGCGAGTCTGCGTCGGCTCC	Downstream (DS) region of *hts hdrB* operon
RL52	ATGAGCGGCGAGGAGCTTTCTGG	Preparation of the DIG-labeled probe
RL53	TTACTCATCGGATTCCTCCG	Preparation of the DIG-labeled probe

Supplementary Table S2. Generation times of *H. volcanii* strains (in hours).

Pairwise measurements	*rpa2*$^{+}$	*gfp*$^{+}$::*rpa2*$^{+}$
1	2.08	2.87
2	1.81	1.87
3	1.42	1.90
4	1.65	2.37
5	2.49	2.13
average ± SD	1.9 ± 0.4	2.2 ± 0.4
